# The spectrum of paediatric cardiac disease presenting to an outpatient clinic in Malawi

**DOI:** 10.1186/1756-0500-6-53

**Published:** 2013-02-07

**Authors:** Neil Kennedy, Paul Miller

**Affiliations:** 1 Department of Paediatrics and Child Health, University of Malawi, P/Bag 360 Chichiri, Blantyre 3, Malawi; 2 Consultant Paediatric Cardiologist, Birmingham Children’s Hospital, Birmingham, UK

**Keywords:** Rheumatic, Global child health, Africa

## Abstract

**Background:**

As progress is made towards attaining Millennium Development Goal 4, further reductions in paediatric mortality will only be achieved by concentrating on the burden of non-communicable or neglected diseases. The literature relating to paediatric cardiac disease in sub-Saharan Africa is sparse. There are no published descriptions of paediatric cardiac disease from Malawi, making it impossible to estimate the contribution it makes to childhood morbidity and mortality.

**Findings:**

In 2008, a paediatric cardiac clinic with echocardiogram scanning was established in Blantyre, southern Malawi. Between January 2009 and February 2011, the age and cardiac diagnosis of every child with an abnormal echocardiogram was recorded in a database. Of 250 children, 139 (55.6%) had congenital heart disease, and 111 (44.4%) acquired heart disease. Ventricular septal defect (VSD) (24%), Tetralogy of Fallot (10%) and patent ductus arteriousus (7.2%) were the commonest forms of congenital heart disease. Rheumatic heart disease (RHD) (22.4%) and dilated cardiomyopathy (13.6%) were the commonest acquired diseases. The mean age of presentation was 3 years 2 months for VSD and 11 years 6 months for RHD.

**Conclusions:**

In this cohort of children from one centre in Malawi, acquired heart disease – in particular rheumatic heart disease was almost as common as congenital heart disease. Most presented late. It is likely that untreated cardiac disease causes a large number of childhood deaths in Malawi. In addition to renewing secondary preventative efforts against rheumatic heart disease, adequate and accessible cardiothoracic surgical services should be established at a regional level.

## Findings

### Background

In Malawi specialised services for children with heart disease are inadequate. This is not surprising given the overwhelming burden of infectious disease. Health services in resource-poor countries such as Malawi have struggled to deal with very ill children suffering from malaria, HIV, pneumonia, malnutrition and diarrhoeal disease [1].

However as good progress in Malawi [1] is made towards achieving millennium goal 4 (‘reducing by 33% the number of children dying before their 5th birthday’), it is increasingly recognised that further sustained reductions in under-5 mortality will only be achieved by concentrating on the burden of non-communicable or neglected diseases [2].

Outside of South Africa, there are few publised studies describing the spectrum or prevalence of paediatric cardiac disease from sub-Saharan Africa [3-7]. The work that exists highlights the untreated cohort of children with congenital heart disease and the additional significant burden of acquired heart disease including rheumatic heart disease [7] and dilated cardiomyopathy. There are no published reports from Malawi.

A small paediatric cardiac clinic with access to echocardiography in Blantyre (Malawi’s largest city and commercial capital) was established in 2008. The aim of this study was to describe the spectrum of cardiac disease presenting to this clinic.

## Methods

The study was performed in Queen Elizabeth Central Hospital (QECH), Blantyre, Malawi. QECH is the largest hospital in Malawi, and the only teaching hospital for undergraduate and postgraduate students. Malawi is one of the poorest countries in the world, with only one trained doctor per 50,000 people [8]. As well as offering secondary care services for Blantyre, a city of one million people, QECH provides tertiary care for the entire Southern Region of Malawi. In 2008, a weekly paediatric cardiac clinic with access to echocardiogram scanning was established in QECH. This is the only clinic of its kind in the country. Children were referred to the clinic for investigation after the diagnosis of structural heart disease was considered in QECH or a district hospital.

Between January 2009 and February 2011, the age and cardiac diagnosis of every child with an abnormal echocardiogram presenting to the paediatric cardiac clinic was recorded in a database (Additional file 1 – QECH paediatric cardiology clinic database).

The diagnosis was established using a portable scanner – the Sonosite 180 with a C11/7-4 Mhz probe. All of the scans were performed by one or both of the authors.

The range of diagnoses of those with an abnormal echocardiogram was described using simple descriptive statistics.

Ethical approval for publication of this data was obtained from the College of Medicine Research Ethics Committee, the local institutional review board.

## Results

Between January 2009 and February 2011, an abnormal echocardiogram was described in 250 children who were seen in the paediatric cardiac clinic. 139 (55.6%) had congenital heart disease, and 111 (44.4%) acquired heart disease. 98 of 250 were female and the median age of presentation was 56 months (range 1–188 months). The spectrum and frequency of diagnoses is shown in Table 1.

**Table 1:** Spectrum of diagnoses

**Diagnosis**	**Number** **(n= 250)**	**Percentage of total**
**CONGENITAL HEART DISEASE**		
Ventricular septal defect	60	24.0
Tetralogy of Fallot	25	10.0
Patent ductus arteriosus	18	7.2
Atrioventricular septal defect	13	5.2
Other cyanotic congenital heart disease	11	4.4
● Transposition with VSD or variant – 3		
● Double outlet right ventricle – 5		
● Other – 3		
Atrial septal defect	5	2.0
Pulmonary stenosis	3	1.2
Aortic stenosis	2	0.8
Coarctation of the aorta	1	0.4
Marfan’s syndrome with aortic regurgitation	1	0.4
**AQUIRED HEART DISEASE**		
Rheumatic heart disease	56	22.4
Dilated cardiomyopathy	34	13.6
Other cardiomyopathy	8	3.2
● Duchenne – 1		
● Hurler’s – 2		
● Mitochondrial – 1		
● Hypertrophic cardiomyopathy – 1		
● Discompacted left ventricle – 3		
Endomyocardial fibrosis	5	2.0
Pericarditis	5	2.0
● Acute tuberculosis – 2		
● Constrictive – 2		
● Pneumococcal – 1		
Secondary pulmonary hypertension	3	1.2
● Upper airway obstruction – 1		
● HIV infected – 1		
● Schistosomiasis – 1		

Of the children with rheumatic heart disease (n= 56), all had mitral regurgitation. In addition, 5 had mitral stenosis, 2 had aortic regurgitation and 1 had tricuspid valve involvement.

The HIV status was known in 22 of those with dilated cardiomyopathy (n=34). 3 children were HIV infected.

The mean age (standard deviation) of presentation to the clinic in months for the commoner diagnoses was as follows:

● Ventricular septal defect 38.0 (42.9)

● Tetralogy of Fallot 51.8 (44.2)

● Dilated cardiomyopathy83.0 (47.7)

● Rheumatic heart disease 137.8 (30.4)

During this period 40 of this cohort of children were referred for corrective surgery (funded mainly by the Ministry of Health in Malawi) in South Africa. By mid 2011, 24 of these children had had an operation.

## Discussion

The spectrum of cardiac disease presenting to the only paediatric cardiology clinic in Malawi is similar to that in other countries from the region [3-7], and stands in stark contrast to that in industrialised nations [9]. In particular, the burden of acquired heart disease is substantial, and the age of presentation very late.

This study has several limitations. Firstly it reports a biased sample of children attending a tertiary level paediatric outpatient clinic in a country where transport links are difficult and many children are not referred. Furthermore children presenting as in-patients referred from other hospitals were excluded as records were unfortunately not available for them. These deficiencies resulted in a relatively small number of children included in the study and grossly underestimate the actual numbers of children with heart disease in the wider community. Nevertheless, given that over 80% of the participants came from a small area - within 10km of the hospital in urban Blantyre- it does provide some valuable information about the range of paediatric cardiac disease that would be found in Malawi if adequate cardiology services existed.

In this study, acquired heart disease comprised 44.4% of the diagnoses made, mostly rheumatic heart disease (RHD). 22.4% of all the children, almost as many as those with ventricular septal defect (VSD), had RHD. If one assumes that approximately 1% of all children in any given population will have congenital heart disease [10], and that the proportion of rheumatic to congenital heart disease in this study is representative of the wider population, then at least 0.4% of Malawi’s children will have acquired rheumatic heart disease. As often only the most severely ill children were referred to this tertiary clinic, it is probable that the prevalence of RHD is much higher than this. In Ethiopia, RHD made up over half of the diagnoses in a paediatric cardiac clinic [6]. An echo-based screening study of schoolchildren in Mozambique estimated the prevalence of RHD at over 3% [7]. A wider population based prevalence survey is urgently required in Malawi. Untreated over 70% of children with established rheumatic heart disease will die by the age of 25 [11]. Therefore it is very probable that acquired heart disease –in particular rheumatic heart disease – makes a significant contribution to childhood and early adulthood morbidity and mortality in Malawi. Efforts at improving access to secondary prevention using regular benzathine penicillin should be renewed [11].

The other commonly occurring acquired disease was dilated cardiomyopathy. Unfortunately, lack of diagnostic investigations precluded more specific diagnoses. However only 3 of 22 patients whose HIV status was known were HIV infected. This represents only 1.2% of the entire cohort. As HIV is known to be associated with significant rates of cardiac disease [12], this suggests that cardiac disorders are not being screened for or recognised in HIV positive children in Malawi.

The age of presentation for both congenital and acquired heart disease was late. The mean age of presentation for a VSD was 3 years and 2months; for rheumatic heart disease it was 11 years and 6 months. It is possible that this is because when the clinic began it was ‘catching up’ on children who were suspected of having heart disease but in whom no diagnosis had been confirmed. However it is more likely that it reflects the paucity of medical services at primary, secondary and tertiary level in Malawi. Many children had been misdiagnosed and treated for recurrent respiratory tract infections or TB rather than cardiac failure. Malawi has less than 20 trained paediatricians for a population of over 14 million, half of whom are children. This scarce resource needs to be used carefully, and outreach services away from the tertiary centres in surrounding district hospitals need to be developed [10].

Of the children with congenital heart disease, the spectrum of conditions in our clinic is similar to that of other African studies [3-6]. The conditions that presented are those that allow survival over the first year of life. This is similar to a study from Zimbabwe which reported an underrepresentation of conditions with a high mortality in infancy [5]. Children with conditions such as transposition of the great arteries do not survive. Many of these early infant deaths are mistakenly attributed to pneumonia or ‘sepsis’, hence underestimating the true burden of cardiac disease and its effects in Malawian children.

This study demonstrates the need for better access to corrective surgery for Malawian children. Apart from patent ductus arteriosus (PDA) ligation there is no cardiothoracic surgical service in Malawi. Therefore corrective surgery for the majority of these children was either unavailable or they presented too late for surgery to be helpful. Many of the children with congenital left to right shunts already had pulmonary hypertension by the time the diagnosis was made. In addition for those with rheumatic heart disease valve replacement or valve repair is hampered by the local unavailability of anti-coagulation therapy. If untreated, nearly one third of children with congenital heart disease will die in the first year of life [10]. Given the additional high mortality of untreated rheumatic heart disease as many as 1-2% of all Malawian children may be dying because of the lack of appropriate diagnostic and surgical services for cardiac disease. We agree with Hewitson and Zilla [10] that there needs to be wider regional coordination of effort to provide an accessible network of surgical centres in Sub-Saharan Africa.

## Conclusions

In this cohort of children from one centre in Malawi, acquired heart disease in particular rheumatic heart disease was almost as common as congenital heart disease. Most present late. A population based prevalence study is required to determine the full extent of this problem. It is likely that untreated cardiac disease causes a significant number of childhood deaths in Malawi. In addition to renewing secondary preventative effort s against rheumatic heart disease, adequate and accessible cardiothoracic surgical services need to be established at a regional level.

### Availability of supporting data

The data set supporting the results of this article is included within the article (and its Additional file – QECH paediatric cardiology clinic database).

## Abbreviations

HIV: Human immunodeficiency virus;PDA: Patent ductus arteriousus;QECH: Queen Elizabeth Central Hospital;RHD: Rheumatic heart disease;VSDm: Ventricular septal defect

## Competing interests

The authors declare that they have no competing interests.

## Authors’ contributions

NK conceived of the study, carried out echocardiograms, performed the analysis and drafted the article. PM carried out echocardiograms. All authors read and approved the final manuscript.

## Authors’ information

NK is a consultant paediatrician at QECH and an Associate Professor of Paediatrics and Child Health at the University of Malawi. PM is a consultant paediatric cardiologist at Birmingham Children’s Hospital which has a long-standing partnership with QECH.

## Supplementary Material

Additional file 1 (QECH Paediatric Cardiology Clinic Database). Click here for file
